# Patterns and Correlates of Physical Symptoms among People with Peripheral Neuropathy

**Published:** 2022-11-29

**Authors:** Miyong T Kim, Nicole Murry, Jacklyn Hecht, Tara Hutson, Tonychris Nnaka, Tiffany Ewere, Elizabeth Heitkemper, Emily T Hebert, Kavita Radhakrishnan, Alexa Stuifbergen

**Affiliations:** 1 Department of Nursing, University of Texas at Austin School of Nursing, USA; 2 Department of Health Promotion and Behavioral Sciences, UT Health Houston School of Public Health, USA

**Keywords:** Peripheral neuropathy, Pain, Numbness, Tingling, Muscle weakness

## Abstract

**Background:**

As the population ages and more people are affected by multiple chronic conditions, the prevalence of Peripheral Neuropathy (PN) has also rapidly increased. This swift rise in PN leaves clinicians and patients challenged by a lack of consistent diagnosis and treatment guidelines.

**Purpose:**

To assist those affected by PN, it is imperative to understand the breadth of symptoms, experiences, and factors related to the quality of life. The primary aims of this study are to (1) characterize the symptoms of PN in a nationwide sample; (2) discern differences in symptom clusters, given perceived causes of PN; and (3) identify significant physiological symptoms related to the quality of life for people with PN.

**Methods:**

An online survey of people in online PN support groups. Participants were recruited primarily *via* an open request sent to recipients of web-based communications from nationally recognized online PN support groups. Inclusion criteria were as follows: Self-reported diagnosis of PN, ability to read English or Spanish, residence in the U.S., and age ≥ 18 years.

**Results:**

A total of 608 individuals with PN were included in the analysis. This sample represents 49 U.S. states and the District of Colombia; 221 were male and 387 female. Their disease severity and etiology were similar to previously reported information on this population, with 53.3% of respondents suffering from PN without a known cause. Among known causes, diabetes was the most common (19.6%), followed by chemotherapy (6.9%) and autoimmune diseases (3.6%). Factors affecting the quality of life among people with PN included lower extremity mobility, upper extremity mobility, sleep disturbance, depressive symptoms, and patient activation.

## Introduction

Peripheral Neuropathy (PN) is a highly prevalent neurological condition typically characterized by symptoms of pain, numbness or tingling, balance issues, and mobility problems caused by damage or injury to the peripheral nervous system [1]. This damage can result in an interruption, disruption, or distortion of signals transmitted from the Central Nervous System to other parts of the body, manifesting in clinical symptoms. Common symptoms of motor nerve damage are muscle weakness, twitching under the skin, and muscle shrinking. Sensory nerve damage can result in pain and numbness. Autonomic nerve damage may lead to excessive sweating, heat intolerance, or difficulty in eating or swallowing [2]. Owing to the heterogeneity of symptoms and multiple etiologies for PN, there is a lack of consistent guidance for PN diagnosis and symptom management.

The estimated number of people with PN in the U.S varies widely, ranging from 16 million to 30 million [2–4] and reflecting a lack of consensus on diagnosis and treatment guidelines. A small proportion of PN diagnoses are associated with genetic disorders [4], but most PN is associated with chronic conditions such as diabetes, cardiovascular disease, autoimmune disease, infection, or cancer. Addressing the root causes of PN and reversing the disease’s progression can improve symptoms. For many people, however, PN is idiopathic, with no known cause. Treatment recommendations exist for diabetes related PN or chemotherapy related PN [5,6]. Still, for those with idiopathic PN or PN with less common causes, healthcare providers tend to focus on managing day to day PN symptoms. The lack of consensus regarding treatment may be related to a lack of data on the symptoms and experiences of those living with PN.

Compared with chronic diseases such as diabetes or high blood pressure, the diagnosis of PN is difficult because of a lack of clear biomarkers to measure damage in the peripheral nervous system. Nerve conduction velocity tests and electromyography have been available for a long time, but we lack a definite biomarker for PN [7]. PN diagnosis and treatment are often secondary to and dependent on the disease’s primary source, even though peripheral nerve channels are anatomically different from affected body parts. When primary conditions and genetic causes are eliminated, PN is classified as idiopathic; occurring in 10% to 50% of cases [2–4]. Despite recommended medical evaluations and tests among people with predominantly sensory alterations that are often progressive, 31% remain idiopathic [8], with treatment standards for PN not yet available [9].

As with other chronic conditions, self-management plays an important role in the illness trajectory and quality of life of people with PN, especially among people with PN and other chronic conditions such as diabetes and high blood pressure. Several studies have explored the effectiveness of self-management programs for people with PN-but most have been part of more extensive studies on primary conditions such as HIV/AIDS [10], cancer [11], and diabetes [5], in which people with idiopathic PN were, of course, excluded. In addition, these studies suffer from small sample sizes and less rigorous designs and the clinical utility of their findings is, therefore, limited [12].

In this study, we present a systematic investigation of people with PN in a national sample, to characterize the main signs and symptoms of PN by etiology and to explore the relationships between PN and both social determinants of health and psychophysiological factors related to self-management. Our findings will inform future interventions, including a contextually relevant self-care support program for people with PN and their families.

## Methods

### Study design

In this study, we used a cross-sectional survey design. The survey was administered online, and data were collected from September 10, 2021, to November 15, 2021. Inclusion criteria were as follows:

- Self-reported diagnosis or symptoms of PN in the past
- Age ≥ 18 years at the time of the survey
- Residence in the U.S.
- Ability to read either English or Spanish
- Willingness to complete the entire questionnaire.

### Sample and recruitment

A random sample at the national level was not feasible because we could not access participants directly to confirm their eligibility. This is a common problem with online surveys [13]. To recruit a national sample, we used strategies for community engagement, such as collaboration with reputable online PN support groups. First, a Community Advisory Board (CAB) consisting of the study’s principal investigator, co-investigators, patients and families, PN support group leaders, and a local neurologist was formed to guide study procedures, including oversight of recruitment. The CAB reviewed questionnaires and recruitment materials (e.g., pamphlets, and program webpages) and provided feedback.

Online recruitment of people with PN was both direct and indirect. Direct recruitment included the development and distribution of targeted recruitment materials shared with established neuropathy support networks the Foundation for Peripheral Neuropathy, the Western Neuropathy Association, and partner agencies. CAB members from our partner organization provided contact information for online support group leaders. The research team contacted 10 support group leaders and sent them a brief study description with a link to our website and survey. The leaders forwarded the survey link to their group members and encouraged them and their friends with PN to participate. Indirect recruitment included posting materials *via* online neuropathy specific Facebook groups with the permission of online group moderators. At the time of the study announcement, the FPN online newsletter had about 30,000 subscribers and the Facebook group “Peripheral Neuropathy Success Stories!” had about 20,000 followers.

Upon completing the survey, participants were offered a $ 25 electronic (Tango) gift card that could be used at multiple local and national vendors and organizations. In addition, participants could request additional resources about neuropathy self-management and choose whether they would like to be contacted again to participate in a future group intervention.

### Measures

We collected demographic information, including zip codes, age, gender, race, ethnicity, language, income, educational attainment, marital status, housing type, employment, living arrangement, and type of healthcare provider (e.g., physician, nurse practitioner, chiropractor, acupuncturist, or other). Physiological data included height and weight. We also gathered data on the cause of PN symptoms and the year PN symptoms began. In addition, we collected extensive data on the participants’ current experience of PN symptoms. Questions included the presence, frequency, timing, and location of pain, numbness, tingling, and muscle weakness. Participants reported these symptoms using the body heat map in which the specific body parts were graphically identified by (x, y) coordinates of each body part including the upper and lower arms and legs, and chest and abdomen. Participants were also asked an open ended question about any additional symptoms they were experiencing.

Other measures were as follows: Neuropathic pain was measured with the Neuropathy Pain Scale (NPS). The NPS is an 11 item scale for pain intensity (including sharpness, sensitivity and itchiness, among others) with 0 indicating less symptom severity and 10 indicating the highest pain symptom severity [14].

Mobility was measured with two parts from the Neuro-QOL, the Lower Extremity Function scale (mobility; 8 items) for Lower Extremity Mobility (LEM) and the Upper Extremity Function scale (fine motor, activities of daily living; 15 items) for Upper Extremity Mobility (UEM). Both scales consist of Likert-type items with responses ranging from 1 (“Unable to do”) to 5 (“Without any difficulty”) [15].

PN related quality of life was measured with the Medical Outcomes Study 12 item Short Form (SF-12). The SF-12 has a physical component summary and a mental component summary, each scored on a scale from 0 to 100. Higher scores represent better physical and mental well-being [16].

Mental health was measured using the Patient Health Questionnaire (PHQ-9). This questionnaire asks about depressive events and their severity in the last week. The scale includes 9 Likert-type items with responses ranging from 0 (“not at all”) to 3 (“nearly every day”). Higher scores indicate higher levels of depressive symptomatology [17].

Patient activation was measured using the Patient Activation Measure (PAM). This unidimensional, Guttman-style questionnaire uses 13 items to assess patients’ knowledge, skills, and confidence about managing their health conditions [18].

Self-management behaviors were measured with a modified High Blood Pressure Self Care Profile (HBP-SCP). This scale includes questions about the frequency of participating in healthy behaviors such as physical activity, eating nutritiously, sleeping adequately, and avoidance of alcohol but also includes questions on mindfulness and spirituality [19].

Sleep quality was measured using the Neuro-QOL Sleep Disturbance scale [15]. This questionnaire has 8 Likert-type items asking about the presence of disturbing events during sleep, daytime sleepiness, and trouble falling asleep. The responses range from 1 (“never”) to 5 (“always”).

Electronic health resource literacy, which describes how a person locates and uses health information on the web, was measured with the eHEALS instrument [20]. An example of a question from this scale is “I know where to find helpful health resources on the internet.” The scale has 8 Likert-type statements with responses ranging from 1 (“Strongly agree”) to 5 (“Strongly disagree”).

### Data analysis

In addition to descriptive statistics for the overall sample and study variables, comparisons between known and unknown causes of PN (known cause vs. idiopathic PN) were explored using x^2^. Bivariate correlations were examined to understand associations among study variables. All analyses were performed with IBM SPSS Statistics 25.0

## Results

### Sample selection

**Figure 1.**
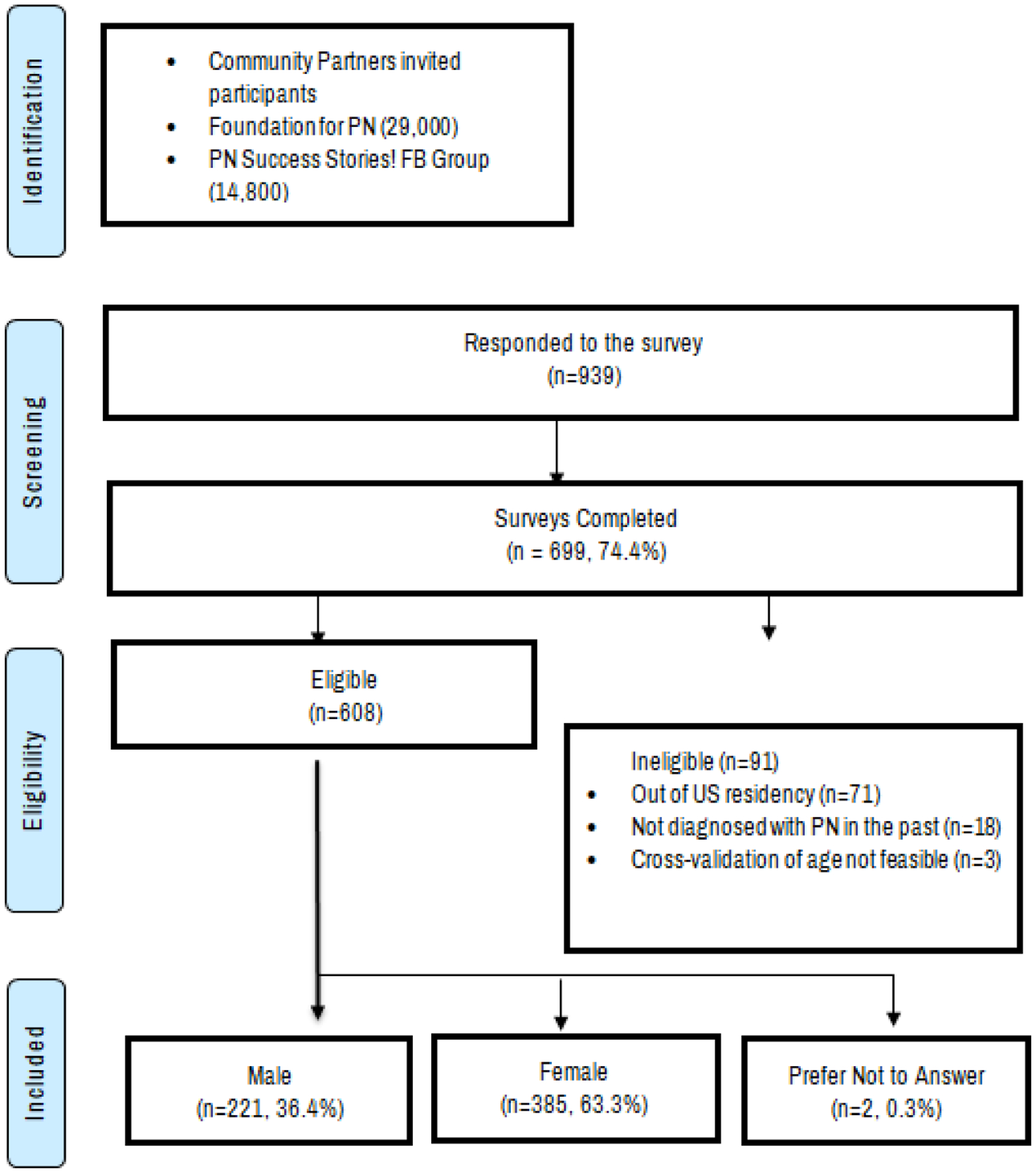
Consort diagram for peripheral neuropathy descriptive study.

A total of 939 people responded to the online survey. After removing people who did not meet the inclusion criteria, complete the survey, or sign the electronic consent form, 608 people remained for the final analysis (Figure 1).

### Demographic characteristics

Nearly two-thirds of those with PN were female (63.3%); two persons chose not to answer. The majorities (81.6%) were aged ≥ 50 years, with a mean age of 63.0 (range, 24 to 94). They were from 49 states in the U.S. Participants were primarily White (94.4%; 13.5% self-identified as Hispanic or Latino), retired (51.8%), highly educated (68.9% graduated from college or higher), residing with one or more persons (77.9%), and making more than $ 50,000/year (54.9%, with 29.0% making more than $ 100,000).

Proportionally, females reported that they were disabled more than males (9.6% vs. 5.4%; difference=4.2%, p<0.001). Males lived more often with another person (62.0% vs. 50.9%; difference=11.1%, p<0.05), and, on average, had PN longer (12.2 vs. 9.0 years; difference=3.2 years, p<0.01) (Table 1).

Of the 608 participants, 46.6% (n=283) reported a known cause for their PN; 53.5% (n=323) reported that the cause was unknown. There were significant differences in demographic characteristics between the two groups. On average, the known cause group was younger (58.4 years) than the unknown cause group (67.0 years; difference=−8.6 years, p<0.001). More individuals in the known cause group than in the unknown cause group were employed full-time (46.3% vs. 26.0%; difference=20.3%, p<0.01), and fewer in the known cause group were retired (37.8% vs. 63.8%; difference=−26%, p<0.001).

There were also differences in educational attainment: The known cause group included more college graduates than did the unknown cause group (37.5% vs. 27.9%, difference=9.5%; p<0.05) but fewer with advanced degrees (29.7% vs. 43.0%; difference=−13.4%, p<0.001). Similarly, the known cause group included fewer high income earners (making more than $ 100,000 a year) than did the unknown cause group (10.6% vs. 37.6%; difference=−18.1%, p<0.05). The known cause group was more diverse ethnic background than the unknown cause group, which consisted of a primarily homogeneous white sample (67.1% vs. 93.8%; difference=−26.7%, p<0.001). More individuals in the known cause group than in the unknown cause group had a physician (MD) as their current medical care provider (78.4% vs. 70.0%; difference=8.4%, p<0.05) (Table 2).

### History and symptoms of peripheral neuropathy

Study participants reported a diagnosis of PN or having PN symptoms for an average of 9.7 years (SD=9.7; range<1 to 54 years). About half (53.3%) reported not knowing the cause of their PN (idiopathic). Among known causes, diabetes was the leading cause (19.6%), followed by chemotherapy (6.9%), autoimmune diseases such as chronic inflammatory demyelinating polyradiculoneuropathy or Sjogren’s syndrome (3.6%), vitamin B12 deficiency (1.2%), and spinal cord surgery (0.8%).

Most (82.5%) had PN symptoms (sensory disturbance or pain) more than once daily; 4.8% had episodes once a day. There were no statistically significant differences in the frequency of PN episodes between males and females. Once the sensory related symptoms manifested, they lasted a few days or longer (58.5%), followed by a few hours (22.4%) and a few minutes (17.6%).

Among symptoms in the last month, the tingling was most prevalent (92.2%), closely followed by a numbness (91.4%), pain (90.3%), and muscle weakness (72.3%). Numbness was felt most in the feet (81.2%), followed by the hands (50.5%) and legs (42.1%), and the least felt in the upper arms (16.7%). Tingling was felt in the same order as numbness, indicating that numbness and tingling might appear together. Muscle weakness was felt mostly in the legs (49.3%), whereas pain was most common in the feet (76.6%) (Table 3).

Males had experienced PN longer than females (M=11.2 years, SD=9.8, vs. M=9.0, SD=9.3; difference=2.2, p<0.01), and they reported fewer tingling sites (4.5 vs. 5.2 sites; difference=−0.7, p<0.01) and less tingling in the feet (71.5% vs. 80.3%; difference=−8.8%, p<0.05).

The differences between people with PN with known causes and people with idiopathic PN were more complicated than those between males and females: For the last month, the known cause group, in comparison with the unknown cause group, reported more symptoms of numbness (94.0% vs. 89.2%; difference=4.8%, p<0.05), muscle weakness (82.6% vs. 63.1%; difference=19.5%, p<0.001), and pain (92.8% vs. 88.1; difference=4.7%, p=0.065). However, the unknown cause group suffered from more frequent PN symptom episodes (“More than once a day,” 72.8% vs. 91.0%; difference=−18.2%, p<0.001), with the episodes longer in duration (“A few days or longer,” 50.9% vs. 64.9%; difference=−14.0%, p<001) and time (“A few minutes,” 27.8% vs. 9.0%; difference=18.8%, p<0.05).

Overall, the known cause PN group reported greater intensity of symptoms than did the idiopathic PN group: Muscle weakness (4.2 sites vs. 3.3 sites; difference=0.9 sites, p<0.001), numbness in areas other than extremities (35.3% vs. 21.1%; difference=14.2%, p<0.05), muscle weakness in the upper arms (32.5% vs. 12.1%; difference=20.4%, p<0.05), pain in the legs (46.3% vs. 32.2%; difference=14.1%, p<0.05). However, the idiopathic group reported more tingling in the feet (81.4% vs. 72.4%; difference=−9.0%, p<0.05) and more foot pain (80.8% vs. 71.4%; difference=−9.4%, p<0.05). On the Neuropathy Pain Scale (range: 0–100), the known cause group reported more severe pain than did the unknown cause group (M=47.5, SD=22.0 vs. M=40.0, SD=21.8; difference=7.4, SE=1.8, p<0.001).

### Correlates of PN episodes and pain severity

Several factors, including the mobility scores of upper extremities and lower extremities, sleep quality, mental health, patient activation, and motivation, were significantly correlated with neuropathy pain total score (p<0.001). So were health behaviors (p<0.05). eHealth literacy skills were not significantly correlated. In addition, there were statistically significant differences between the cause known group and the idiopathic group in the PN episodes. The cause known group reported more PN episodes in the upper extremities (p<0.01), better sleep quality but less mental health (p<0.05), more coping skills (p<0.001), but fewer eHealth skills (p<0.01).

The cause known group also reported more severe pain expressions except for unpleasantness, not statistically significant: The differences in sharpness, dullness, coldness, sensitiveness, and itchiness were statistically significant.

### Qualitative data indicating unmet needs of people with PN

Participants were invited to offer additional comments at the end of the survey, and 361 provided written responses. The most common themes are summarized, along with sample statements in participants’ own words.

Although some participants reported mild to moderate life disruption, many described deep suffering and despair: “It is hell living with this disease. I hope and pray for a cure”; “It’s lonely agony”; “I am very scared about my future.” Participants also commented on the lack of information and the impact of uncertainty on their outlook: “Anxiety occurs due to the uncertainty of the disease progression/remission and not knowing when flare-ups will occur.” They also lamented the medical community’s lack of knowledge and investment in finding a cure or better treatment options: “I don’t think the medical community is interested in a cure or finding the cause. I no longer waste money and time with doctors. Neuropathy FB (Facebook) groups and research keep me abreast…” Finally, they shared self-management approaches that helped them cope with physical and psychological symptoms: “At first I was down about that situation but in the past few years I have completely accepted my condition and found physical activities like golf, biking, some walking and in particular water aerobics and swimming to fill the need I have to stay fit and active” (Table 4).

## Discussion

The findings of this descriptive study are consistent with prior literature, and they suggest areas for further research on PN. The prevalence of idiopathic PN (over 50% of those with PN) is noteworthy. Clinical research has predominantly focused on diabetes related PN, and our understanding of less common types and causes of PN is limited. Several plausible metabolic related physiological pathways for diabetes related PN have been proposed, and treatments for severe symptoms such as pain from diabetes related PN are available [21]; but treatment options for other types of PN are scarce. The inability to treat this growing patient group is a serious public health concern as the population ages and the prevalence of idiopathic PN increases [2,3]. Moreover, the participants’ qualitative data indicated that they were frustrated with the “lack of clear diagnosis and treatment guidelines” and inadequate “sensible self-management support” for their conditions. Given the chronic nature of these conditions, the need for evidence based self-management support is paramount. The heterogeneity of symptom manifestation and differences in perceived quality of life among those with known and unknown causes of PN suggest that basic research on different physiological pathways and on the role that precision medicine could play in addressing these deficits is warranted [22].

Our analysis revealed that there were minimal differences in symptoms by gender, although more females reported being “disabled” than males, whereas more males reported suffering from PN symptoms. On the other hand, analysis by known cause versus the unknown cause of PN demonstrated significant differences in demographic characteristics. The idiopathic group reported older age and higher socioeconomic status in comparison with people with known etiologies of PN, and they were predominantly White.

These findings, however, need to be interpreted with caution, given the limitations of our sample’s characteristics. This was a predominantly White sample, and data were collected online. Our analysis of the National Health and Nutrition Examination Survey (NHANES) of 3 cycles between 1999–2004 indicated that PN using the monofilament test was more prevalent in racial minorities. When each racial group’s PN prevalence was compared to its proportion of the population in the nation, Black was proportionally the highest prevalence (15.1% PN vs. 9.1% national population composition), followed by others (5.2% vs. 3.8%), Mexica American (5.0% vs 4.5%), and Hispanic (5.4% vs. 6.8%). Whites were 67.8% of PN cases, which was lower than the 77.3% of the national population composition. Women were oversampled in our sample, compared to the above national sample (66.3% vs. 54.0%). With the inclusion of diverse groups of racial/ethnic minorities, gender, and socioeconomic status (i.e., social determinants of health, SDH), the results might be different. The PN prevalence may be despair by the SDH. Still, our findings indicate the perceived difference of the PN etiology (i.e., cause known vs. idiopathic) has much stronger correlations with PN episodes and pain severity than the gender difference (i.e., one SDH).

For example, the known etiology group reported muscle weakness and numbness in areas other than extremities, whereas the idiopathic group reported more sensory alteration in the feet and lower extremities. These symptoms require serious attention because sensory alterations in the lower extremes are frequent precursors of serious disease progression and subsequent mobility impairment. Since the quality of life for all PN groups, regardless of etiology, is closely related to LEM, self-management guidelines should include ways to preserve LEM. Other significant quality of life predictors for people with PN include sleep disturbance, depressive symptoms, patient activation, and self-care behavior. Furthermore, due to the heterogeneity of symptom manifestations and differences in perceived quality of life among those with known and unknown causes of PN, basic research should examine different physiological pathways and precision medicine’s role in addressing these deficits [22].

The state of the clinical science of PN indicates that reversing established PN is unlikely because there is no effective pharmacological treatment for PN symptoms, even for those with well-known etiology [23]. In the case of people with diabetes, active treatment of hyperglycemia may offer some prevention or delay in diabetes related PN. Even more aggressive treatment, such as pancreatic transplantation, that may afford some diabetes related PN stability does not lead to PN improvement [24].

Given the chronic nature of conditions related to PN, the need for evidence based self-management support is paramount; lifestyle based strategies such as improving diet, increasing physical activity, and reducing weight consistently show positive results, with some rigorous studies highlighting the potential for enhanced peripheral nerve regeneration. For example, the landmark trial by the National Institute of Diabetes and Digestive and Kidney Diseases Diabetes Prevention Program (DPP, N=3,234) randomized participants with prediabetes to placebo, metformin, or a lifestyle behavioral modification program integrating diet and exercise [25].

In the lifestyle behavior modification group in the DPP, the risk of progression to diabetes during an average follow-up of 2.8 years was reduced by 58% compared to the placebo group, and b 31% compared with those taking metformin [26]. Moreover, the Impaired Glucose Tolerance Neuropathy Study [27] implemented a similar lifestyle-based intervention among 32 patients with diabetes related neuropathy. All 32 participants received dietary counseling (targeted weight loss, 7%) and increased weekly exercise of at least 150 minutes for 1 year per the DPP guidelines. The objective outcome measure of Intra-Epidermal Nerve Fiber Density (IENFD) and subjective measures (visual analog pain scales) showed that metabolic improvement was associated with slight nerve fiber improvement. After 1 year of this lifestyle intervention, there was a significant improvement in IENFD through skin biopsy. The improvement in IENFD was significantly correlated with an improvement in neuropathic pain. Qualitative data from our study participants indicated they were frustrated with the “lack of clear diagnosis and treatment guidelines” and the inadequate “sensible self-management support” for their conditions.

This study does have clinical implications. This is a national-level PN study with patient centered information, including patients’ unmet needs. The data show that our healthcare system is not equipped to provide precise, meaningful clinical management or self-management support for many people affected by PN. Given that this population is growing and experiencing significant mental health stressors that impact the quality of life, future research and treatment plans should include more patient-engaged efforts. Despite the notion that the PN population is hard to reach, our study suggests creative ways to partner with the PN community to collect patient outcome data successfully [2,3]. For example, using community based participatory research [28] as our operational framework, we partnered with several patient advocacy organizations to create Project HEALING: Health, Empowerment, and Autonomy and Learning in Neuropathy Groups. Working with reputable community groups as study ambassadors to reach the target population was a fruitful strategy for recruiting patients. Leaders of several online PN support groups enthusiastically shared our study recruitment materials, confirming the need to recognize this debilitating disease and quality information and resources on disease management for people suffering from PN. Thus community based participatory research is effective in rolling out a descriptive assessment for an understudied population. This study’s findings can inform the future collaborative development and implementation of effective PN interventions.

## Conclusion

This study highlights the widespread prevalence of idiopathic PN, pervasive debilitating symptoms and diminished quality of life for all people with PN. That we were able to enroll over 600 people (with minimal incentive) into our survey assessment within 2 months through online PN support groups suggests that people with PN are eager to share their experiences and have their voices heard. The lack of treatment and self-management support for this population further highlights the need for creative, compassionate interventions for those with PN. This may include an emphasis on lifestyle behaviors that support daily functioning and mindfulness approaches to promote wellness and thriving, despite continuing symptoms. Given the lack of adequate clinical treatment, more attention should be paid to community based efforts to support people with PN by focusing on symptom management and activities of daily living.

## Figures and Tables

**Table 1.** Demographic characteristics by gender.

Indicator	Male	Female	Difference (M-F)*	Total
Gender, n (%)	221 (36.4%)	385 (63.3%)	−26.9%^3^	606(100.0%)
Age, years, mean (SD)	64.2 (15.3)	62.5 (13.5)	1.7 (1.2)	63.1 (14.2)
Retired, n (%)	124 (51.6%)	191 (49.6%)	2.00%	315 (51.8%)
Working, n (%)	76 (34.5%)	139 (36.1%)	−1.60%	215 (32.4%)
Disabled, n (%)	12 (5.4%)	37 (9.6%)	−4.2%^3^	49 (8.2%)
Graduating College or more, n (%)	158 (71.5%)	259 (67.3%)	4.20%	419 (69.1%)
**Annual income, n (%)**				
< $ 50,000	58 (27.4%)	125 (33.9%)	−6.50%	183 (31.5%)
$ 50,000-< $ 100,000	75 (35.4%)	154 (41.7%)	−6.30%	229 (39.4%)
$ 100,000 or more	79 (37.3%)	90 (24.4%)	−12.90%	169 (29.1%)
**Family size, n (%)**				
Living alone	34 (15.4%)	100 (26.1%)	−10.70%	134 (22.2%)
Two	137 (62.0%)	195 (50.9%)	−11.1%^1^	332 (55.0%)
Three or more	50 (22.6%)	88 (23.0%)	−0.40%	138 (22.9%)
**Race, n (%)**				
American Indian	4 (1.8%)	2 (0.5%)	1.30%	6 (1.0%)
Asian American	5 (2.3%)	2 (0.5%)	1.80%	7 (1.1%)
African American	5 (2.3%)	11 (2.9%)	−0.60%	16 (2.6%)
Native Hawaiian or Pacific Islanders	0 (0.0%)	2 (0.5%)	−0.50%	2 (0.3%)
White	169 (76.5%)	325 (84.4%)	−7.9%^1^	494 (81.5%)
Hispanic or Latino	38 (17.2%)	43 (11.2%)	−6.00%	81 (13.4%)
**Cause, n (%)**				
Diabetes	56 (25.3%)	62 (16.2%)	9.10%	118 (19.5%)
Chemotherapy	13 (5.9%)	28 (7.3%)	−1.40%	41 (6.8%)
Don’t know	120 (54.3%)	203 (53.0%)	1.30%	323 (53.3%)
**Medical provider**				
Physician (MD)	173 (78.3%)	275 (71.4%)	6.90%	448 (73.9%)
Nurse practitioner	22 (10.0%)	39 (10.1%)	−0.10%	61 (10.0%)
Chiropractor	10 (4.5%)	20 (5.2%)	−0.70%	30 (5.0%)
Acupuncturist	19 (8.6%)	18 (4.7%)	3.90%	37 (6.1%)
Other	40 (18.1%)	85 (22.1%)	−4.00%	125 (20.6%)
No provider	13 (5.9%)	17 (4.4%)	1.50%	30 (5.0%)

Note:

1 Signifiant at p<0.05;

2 P<0.01;

3 P<0.001;

* SE

**Table 2.** Demographic characteristics by cause.

Indicator	Cause Known (A)	Idiopathic (B)	Difference*(A-B)	Total
Number, (%)	283 (46.6%)	323 (53.4%)	−6.80%	606 (100%)
**Gender, n (%)**				
Male	101 (35.7%)	120 (37.1%)	−1.40%	221 (36.5%)
Female	180 (63.6%)	203 (62.9%)	0.07%	383 (63.2%)
Prefer not to answer	2 (0.3%)	0 (0.0%)	--	2 (0.3%)
Age, years, mean (SD)	58.4 (14.8)	67.0 (12.5)	−8.6 (1.2)^3^	63.0 (14.3)
**Working status, n (%)**				
Retired	107 (37.8%)	206 (63.8%)	−26.0%^3^	313 (51.7%)
Working	134 (46.3%)	84 (26.0%)	20.3%^2^	215 (35.5%)
Disabled	27 (9.5%)	23 (7.1%)	2.40%	50 (8.3%)
**Education level, n (%)**				
Advanced degree	84 (29.7%)	139 (43.0%)	−13.4%	223 (36.8%)
Graduate college	106 (37.5%)	90 (27.9%)	9.5%^1^	196 (32.3%)
Complete some college	59 (20.9%)	55 (17.0%)	3.90%	114 (18.8%)
Graduate high school	32 (11.3%)	37 (11.5%)	−0.20%	69 (11.4%)
**Annual income, n (%)**				
< $ 20,000	22 (8.0%)	22 (7.2%)	0.80%	44 (7.6%)
$ 20,000-< $ 35,000	37 (13.4%)	28 (9.2%)	4.20%	65 (11.2%)
$ 35,000-< $ 50,000	44 (15.9%)	32 (10.5%)	5.40%	76 (13.1%)
$ 50,000-< $ 75,000	77 (27.9%)	57 (18.6%)	9.30%	134 (23.0%)
$ 75,000-< $ 100,000	42 (15.2%)	52 (17.0%)	−1.80%	94 (16.1%)
$ 100,000 or more	54 (19.6%)	115 (37.6%)	−18.1%	169 (29.0%)
**Family size, n (%)**				
Living alone	60 (21.2%)	74 (23.1%)	−1.90%	134 (22.2%)
Two	134 (47.4%)	198 (61.7%)	−14.3%^2^	332 (55.0%)
Three or more	89 (31.5%)	49 (15.3%)	16.2%^1^	138 (22.9%)
**Race, n (%)**				
American Indian	2 (0.7%)	4 (1.2%)	−0.50%	6 (1.0%)
Asian American	6 (2.1%)	1 (0.3%)	1.80%	7 (1.2%)
African American	11 (3.9%)	5 (1.6%)	2.30%	16 (2.6%)
Native Hawaiian or Pacific Islanders	1 (0.3%)	1 (0.3%)	−0.50%	2 (0.3%)
White	190 (67.1%)	303 (93.8%)	−26.7%^3^	495 (81.4%)
Hispanic or Latino	73 (25.8%%)	9 (2.8%%)	23.00%	82 (13.5%)
**Medical provider**				
Physician (MD)	222 (78.4%)	226 (70.0%)	8.4%^1^	448 (73.9%)
Nurse practitioner	38 (13.4%)	23 (7.1%)	6.30%	61 (10.0%)
Chiropractor	14 (4.9%)	17 (5.3%)	−0.40%	30 (5.0%)
Acupuncturist	22 (7.8%)	15 (4.6%)	3.20%	37 (6.1%)
Other	49 (17.3%)	76 (23.5%)	−6.20%	125 (20.6%)
No provider	8 (2.8%)	21 (6.5%)	−3.70%	30 (5.0%)

Note:

1 Signifiant at p<0.05;

2 P<0.01;

3 P<0.001;

* SE

**Table 3.** Peripheral neuropathy symptoms by cause.

Variables	Cause Known (A)	Idiopathic (B)	Difference*(A-B)	Total
PN years, mean (SD)	8.7 (9.3)	10.6 (9.9)	−1.9 (0.7)^1^	9.8 (9.7)
**Cause, n (%)**				
Diabetes 1190	119 (25.3%)	--	--	119 (19.5%)
Chemotherapy	42 (14.8%)	--	--	42 (6.9%)
Other	122 (43.1%)	--	--	122 (20.1%)
Idiopathic		323 (100%)	--	323 (53.3%)
Frequency, n (%)				
A few times a month	46 (16.3%)	5 (1.6%)	14.70%	51 (8.4%)
Once a day	15 (5.3%)	13 (4.0%)	1.30%	28 (4.6%)
More than once a day	206 (72.8%)	294 (91.0%)	−18.2%^3^	500 (82.5%)
**Duration, n (%)**				
A few days or longer	143 (50.9%)	209 (64.9%)	−14.0%^2^	352 (58.4%)
A few hours	56 (19.9%)	79 (24.5%)	−4.60%	135 (22.4%)
A few minutes	78 (27.8%)	29 (9.0%)	18.8%1	107 (17.7%)
**Symptoms in the last month, n (%)**				
Numbness	264 (94.0%)	288 (89.2%)	4.8%^1^	552 (91.4%)
Tingling	259 (91.8%)	294 (92.5%)	−0.70%	553 (92.2%)
Muscle Weakness	233 (82.6%)	200 (63.1%)	19.5%^3^	433 (72.3%)
Pain	258 (92.8%)	280 (88.1%)	4.70%	538 (90.3%)
**No. body parts with symptoms (range: 0–8), mean (SD)**				
Numbness	5.7 (2.5)	5.4 (2.8)	0.3 (0.2)	5.5 (2.7)
Tingling	5.1 (2.7)	4.9 (2.8)	0.2 (0.2)	5.0 (2.7)
Muscle Weakness	4.2 (2.7)	3.3 (3.0)	0.9 (0.2)^3^	3.7 (2.9)
Pain	4.7 (2.4)	4.3 (2.5)	0.4 (0.2)	4.5 (2.5)
**Numbness site, n (%)**				
Upper Arms	73 (25.8%)	28 (8.7%)	17.10%	101 (16.7%)
Hands	158 (55.8%)	149 (46.1%)	9.70%	307 (50.5%)
Legs	132 (46.6%)	123 (38.1%)	8.50%	255 (42.1%)
Feet	173 (78.3%)	319 (82.9%)	−4.60%	492 (81.2%)
Others	100 (35.3%)	68 (21.1%)	14.2%^1^	168 (27.7%)
**Tingling site, n (%)**				
Upper Arms	69 (24.4%)	27 (8.4%)	16.00%	96 (15.8%)
Hands	139 (49.1%)	139 (43.0%)	6.10%	278 (45.7%)
Legs	125 (44.2%)	110 (34.1%)	10.10%	235 (38.8%)
Feet	205 (72.4%)	263 (81.4%)	−9.0%^1^	468 (77.2%)
Others	89 (31.5%)	57 (17.7%)	13.80%	146 (24.1%)
**Muscle weakness site, n (%)**				
Upper Arms	92 (32.5%)	39 (12.1%)	20.4%^1^	131 (21.6%)
Hands	87 (30.7%)	77 (23.8%)	6.90%	164 (27.1%)
Legs	157 (55.5%)	143 (44.3%)	11.20%	300 (49.5%)
Feet	113 (39.9%)	121 (37.5%)	2.40%	234 (38.6%)
Others	83 (29.3%)	78 (24.2%)	5.10%	161 (26.6%)
**Pain sites, n (%)**				
Upper Arms	65 (23.0%)	25 (7.7%)	15.30%	90 (14.9%)
Hands	94 (33.2%)	102 (31.6%)	0.60%	196 (32.3%)
Legs	131 (46.3%)	104 (32.2%)	14.1%^1^	235 (38.8%)
Feet	202 (71.4%)	261 (80.8%)	−9.4%^1^	463 (76.4%)
Others	102 (36.0%)	80 (24.8%)	11.20%	182 (30.0%)
Neuropathy Pain score (range: 0–100), mean (SD)	47.5 (22.0)	40.0 (21.8)	7.4 (1.8)^3^	43.5 (22.0)

Note:

1 Signifiant at p<0.05;

2 P<0.01;

3 P<0.001;

* SE

**Table 4.** Qualitative Comments.

Theme	Participant Quotes
Lack of information and uncertainty	“There is an appalling lack of information or research to find a cure” “There is too much conflicting information about what helps and what doesn’t” “I don’t find any assistance from my doctors. My greatest help has been in alternative medicine” “For idiopathic neuropathy, very little research is being done to discover what causes it.” “Worst is not knowing” “The worst thing is the lack of control and uncertainty”
Emotional Suffering	“It’s miserable” “It’s horrible” “Soul sucking” “It’s a daily struggle and I’ll never be the same.” “…quality of life has deteriorated and depression has become severe.” “It is hell living with this disease. I hope and pray for a cure.” “It’s isolating and you fear ending up all alone”
Physical Limitations and Balance Issues	“Due to pain my activity level has decreased significantly” “Balance problems; constant fear of falling” “Loss of balance due to loss of sensation in my lower extremities”
Pain	“Electric shock type pain in neck and face fairly regularly” “The pain is relentless. It never goes away.” “It is very hard to get and stay motivated when you are in constant pain”
Frustration in Seeking Answers	“How frustrating it is to seek out help for this condition and then experience the treatment made my neuropathy worse! This makes it hard for me to try new approaches” “It would be helpful if there were more support groups based upon science.” “The hardest thing is Dr putting us on meds that don’t help and have horrible side effects”
Helpful Strategies and Self-Advocacy	“Focusing on balance is critical. Nutritional and supplement knowledge is critical in managing PN” “Being proactive in my own health and researching helps me feel in control of my health” “I direct a local PN support group. I think it is critical that we advocate for ourselves” “While I miss my old physical activities, I have found others that can take their place that fit my physical limitation”
